# Association of Epstein-Barr virus serological reactivation with transitioning to systemic lupus erythematosus in at risk individuals

**DOI:** 10.1136/annrheumdis-2019-215361

**Published:** 2019-06-19

**Authors:** Neelakshi R. Jog, Kendra A. Young, Melissa E. Munroe, Michael T. Harmon, Joel M. Guthridge, Jennifer A. Kelly, Diane L. Kamen, Gary S. Gilkeson, Michael H. Weisman, David R. Karp, Patrick M. Gaffney, John B. Harley, Daniel J. Wallace, Jill M. Norris, Judith A. James

**Affiliations:** 1 Arthritis and Clinical Immunology Program, Oklahoma Medical Research Foundation, Oklahoma City, OK, USA; 2 Colorado School of Public Health, University of Colorado Anschutz Medical Campus, Aurora, CO, USA; 3 Department of Medicine, Medical University of South Carolina, Charleston, SC, USA; 4 Division of Rheumatology, Cedar-Sinai Medical Center, Los Angeles, CA, USA; 5 Division of Rheumatic Diseases, University of Texas Southwestern Medical Center, Dallas, TX, USA; 6 Center of Autoimmune Genomics and Etiology, Cincinnati Children’s Hospital Medical Center, Cincinnati, OH, USA; 7 Department of Pediatrics, University of Cincinnati College of Medicine, Cincinnati, OH, USA; 8 US Department of Veterans Affairs Medical Center, Cincinnati, OH, USA; 9 Departments of Medicine and Pathology, University of Oklahoma Health Science Center, Oklahoma City, OK, USA

**Keywords:** Systemic Lupus Erythematosus, Autoimmunity, Epstein-Barr virus

## Abstract

**Objective.:**

Systemic lupus erythematosus (SLE) is a systemic autoimmune disease with unknown etiology. Epstein-Barr virus (EBV) is an environmental factor associated with SLE. EBV maintains latency in B cells with frequent reactivation measured by antibodies against viral capsid antigen (VCA) and early antigen (EA). In this study, we determined whether EBV reactivation and single nucleotide polymorphisms (SNPs) in EBV-associated host genes are associated with SLE transition.

**Methods.:**

SLE patient relatives (n=436) who did not have SLE at baseline were re-contacted after 6.3 (±3.9) years and evaluated for interim transitioning to SLE (≥4 cumulative ACR criteria); 56 (13%) transitioned to SLE prior to the follow-up visit. At both visits, detailed demographic, environmental, clinical information, and blood samples were obtained. Antibodies against viral antigens were measured by ELISA. SNPs in *IL10*, *CR2*, *TNFAIP3*, and *CD40* genes were typed by ImmunoChip™. Generalized estimating equations were used to test associations between viral antibody levels and transitioning to SLE.

**Results.:**

Mean baseline VCA IgG (4.879±1.797 *vs* 3.866±1.795, *p*=0.0003) and EA IgG (1.192±1.113 *vs* 0.7774±0.8484, *p*=0.0236) levels were higher in transitioned compared to autoantibody negative non-transition relatives. Increased VCA IgG and EA IgG were associated with transitioning to SLE (OR 1.28 95%CI 1.07–1.53 *p*=0.007, OR 1.43 95%CI 1.06–1.93 *p*=0.02, respectively). Significant interactions were observed between *CD40* variant rs48100485 and VCA IgG levels, and *IL10* variant rs3024493 and VCA IgA levels in transitioning to SLE.

**Conclusion.:**

Heightened serologic reactivation of EBV increases the probability of transitioning to SLE in unaffected SLE relatives.

## Introduction

Systemic lupus erythematosus (SLE) is a systemic autoimmune disease that involves multiple organ systems and causes significant morbidity. The etiology of SLE is not completely understood. Genetic risk factors have been associated with SLE.[1 ,2] However, monozygotic twin concordance rates of 24–69% [3 ,4] suggest a role for environmental factors in SLE pathogenesis.

Epstein-Barr virus (EBV) is a gamma-herpes virus that ubiquitously infects the majority of the world’s population. EBV maintains latency in B cells and occasionally reactivates the lytic cycle. EBV infection elicits an IgG response to viral capsid antigen (VCA), followed by an IgG response to early antigen (EA). EA IgG levels are detectable as early as a few months and up to 2 years following infection. VCA IgG responses persist for life, although levels are lower during latency compared to lytic infection.[5] Reactivation of EBV is proposed to increase VCA IgG levels and also leads to detectable EA IgG levels.[5] Serology can therefore be used to identify individuals with current infection or recent viral reactivation.

Significant associations between SLE and serologic markers of EBV infection, VCA IgG and EA IgG, have been shown.[6–8] SLE patients have higher frequencies of EBV-infected cells, higher viral loads in blood mononuclear cells, and higher levels of EA IgG,[9–12] suggesting more frequent EBV reactivation. However, whether this recurring EBV reactivation is a consequence of immune dysregulation and/or immune suppression in SLE patients is not clear. To our knowledge, no prospective studies have been performed to understand the cause/effect relationship between viral reactivation and SLE classification. The ligation of human complement receptor 2 (CR2) with gp350/220 glycoprotein of EBV leads to endocytosis and subsequent EBV infection of B cells.[13 ,14] EBV encodes homologues of human proteins to aid in infection and/or maintain latency. An EBV homologue of Interleukin 10 (IL-10) promotes B cell survival and induces pro-inflammatory phenotype in monocytes.[15 ,16] Latent Membrane Protein 1 (LMP-1), a viral mimic of CD40, induces extrafollicular B cell differentiation.[17] EBV infection induced CD40L/CD40 signaling in host cells may support viral persistence.[18] Interestingly, candidate gene studies in SLE patients relative to healthy controls have revealed that single nucleotide polymorphisms (SNPs) in host *IL10* (rs3024505, rs3024495, rs3024493, and 3122605) and *CD40* (rs1569723, rs1883832, and rs4810485), and a three SNP *CR2* haplotype (rs3813946, rs1048971, and rs17615) are associated with lupus susceptibility.[19–23] Relationships between EBV infection/reactivation and genetic variants in host genes implicated in these viral-associated pathways in SLE are unknown.

In this study, we examined whether EBV serological reactivation precedes SLE classification and whether the associations between serologic measures of EBV reactivation and transitioning to SLE were modified by variants in host EBV related genes. Interactions between EBV reactivation measures and SNPs in *TNFAIP3*, which is associated with SLE, but not involved in EBV infection, in SLE transitioning were also determined.

## Methods

### Study design and patients

Study participants were previously enrolled in the Lupus Family Registry and Repository (LFRR) and Systemic Lupus Erythematosus in Gullah Health (SLEIGH) studies [24 ,25] and provided consent for re-contact. This study analyzed baseline samples of individuals (n=436) who had a relative with SLE and who did not meet ≥4 American College of Rheumatology (ACR) criteria for SLE at the time of their baseline visit. were enrolled in aA follow-up study assessed ACR criteria to determine interim transition to classified SLE in these previously unaffected relatives, hereafter referred to as non-transitioned (<4 ACR criteria at follow-up) or transitioned (≥4 ACR criteria at follow-up) relatives. Unrelated unaffected controls, hereafter referred to as controls (n=122), were recruited at local health fairs in Oklahoma and were age- and sex-matched to SLE relatives. All study participants provided informed consent prior to enrollment. All protocols were approved by the Oklahoma Medical Research Foundation (OMRF) or Medical University of South Carolina (MUSC) Institutional Review Boards to be compliant with the Helsinki Declaration.

### Patient and public involvement

Research was done without patient involvement in the study design, data analysis/interpretation, or writing/editing of this manuscript.

### Autoantibody and anti-viral response testing

Autoantibody testing was performed by the CAP-certified/CLIA-approved OMRF Clinical Immunology Laboratory as described previously.[26 ,27] Anti-nuclear antibodies (ANA) and anti-double stranded DNA (dsDNA) antibodies were determined by indirect immunofluorescence using Hep2 cells or *Crithidia luciliae*, respectively (INOVA Diagnostics, San Diego, CA). Positivity was defined as ANA detection at a titre of ≥ 1:120 and anti-dsDNA detection at ≥ 1:30. Antibodies against extractable nuclear antigens (Ro, La, Sm, nRNP, and ribosomal P) were detected by immunodiffusion. Anti-cardiolipin (aCL) antibodies were measured by enzyme-linked immunosorbent assay (ELISA). Positivity was defined as >20 IgG or IgM units.

Antibodies against EBV VCA (IgG), EBV EA (IgG) (Zeus Scientific (Alere-Wampole/Abbott), Branchburg, NJ), and EBV VCA (IgA) (Calbiotech, El Cajon, CA) were measured using commercial ELISAs according to manufacturer’s instructions in samples collected at the baseline visit for SLE relatives and controls. Antibodies against unrelated herpes viruses, Cytomegalovirus (CMV, IgG) and Herpes Simplex Virus-1 (HSV1, IgG), and Varicella zoster (VZV, IgG) (Zeus Scientific (Alere-Wampole/Abbott), Branchburg, NJ) were also measured. Standard calibrators were used in each assay to calculate index values/optical density (OD) ratios, which serve as a semi-quantitative measure of antibody levels. All assays met pre-determined quality control measures based on positive, negative, and blank controls. Positivity was defined as OD ratio ≥1.1.

### Genotyping Study Participants

Five SNPs in *IL10*, three in *CD40*, one in *CR2*, and five in *TNFAIP3*, previously shown to be associated with autoimmune diseases, were genotyped using the ImmunoChip™ and were read on the Illumina iScan in the OMRF Clinical Genomics Center as described previously. [28] Each SNP was tested for consistency with Hardy-Weinberg proportions using a one-degree of freedom χ2 goodness-of-fit test with a *p* value of 0.05 considered as evidence of a departure from Hardy-Weinberg equilibrium. *IL10* variants, rs3024505, rs3024495, rs3024493, rs3122605, were in strong linkage disequilibrium (r^2^≥0.80). Similarly, *CD40* SNPs, rs1569723, rs1883832, rs4810485, were in strong linkage disequilibrium (r^2^>0.80). *IL10* SNP rs3024493 and *CD40* SNP rs4810485 were therefore used as surrogates for other SNPs in *IL10* and *CD40* genes, respectively.

### Statistical analysis

Viral antibody levels at baseline between transitioned relatives (n=56), non-transitioned relatives (n=380), and control (n=122) groups were compared using the Mann-Whitney U test (GraphPad Prism version 7.02). The percent of individuals positive for anti-viral responses in the different groups were compared by Fisher’s exact test for categorical variables (GraphPad Prism version 7.02). Generalized estimating equations (GEE), adjusting for correlation within families, were used to test associations between the viral antibody levels and the categorical outcome of transitioning to SLE. Odds ratios (OR) are reported for one unit change in antibody level (OD ratio). Associations with SNPs were examined in an additive model treating the number of minor alleles as a continuous variable with the OR representing an increase (or decrease) in risk for each minor allele. OR and 95% confidence intervals (CI) were determined for all models. GEE analyses were performed in SAS V9.4. All multivariable models were adjusted for age, sex, and race.

## Results

### Study cohort

Nearly 75% of study participants were of European American descent and more than 85% were female. No significant differences were observed in average age between transitioned and non-transitioned relatives. Transitioned relatives had a greater number of confirmed ACR criteria at study enrollment, and higher percentages were ANA positive at baseline compared to non-transitioned relatives (*p*<0.0001, Table 1).

### Relatives who transitioned to SLE had higher baseline levels of anti-VCA IgG compared to relatives who did not transition to SLE

**Figure 1.**
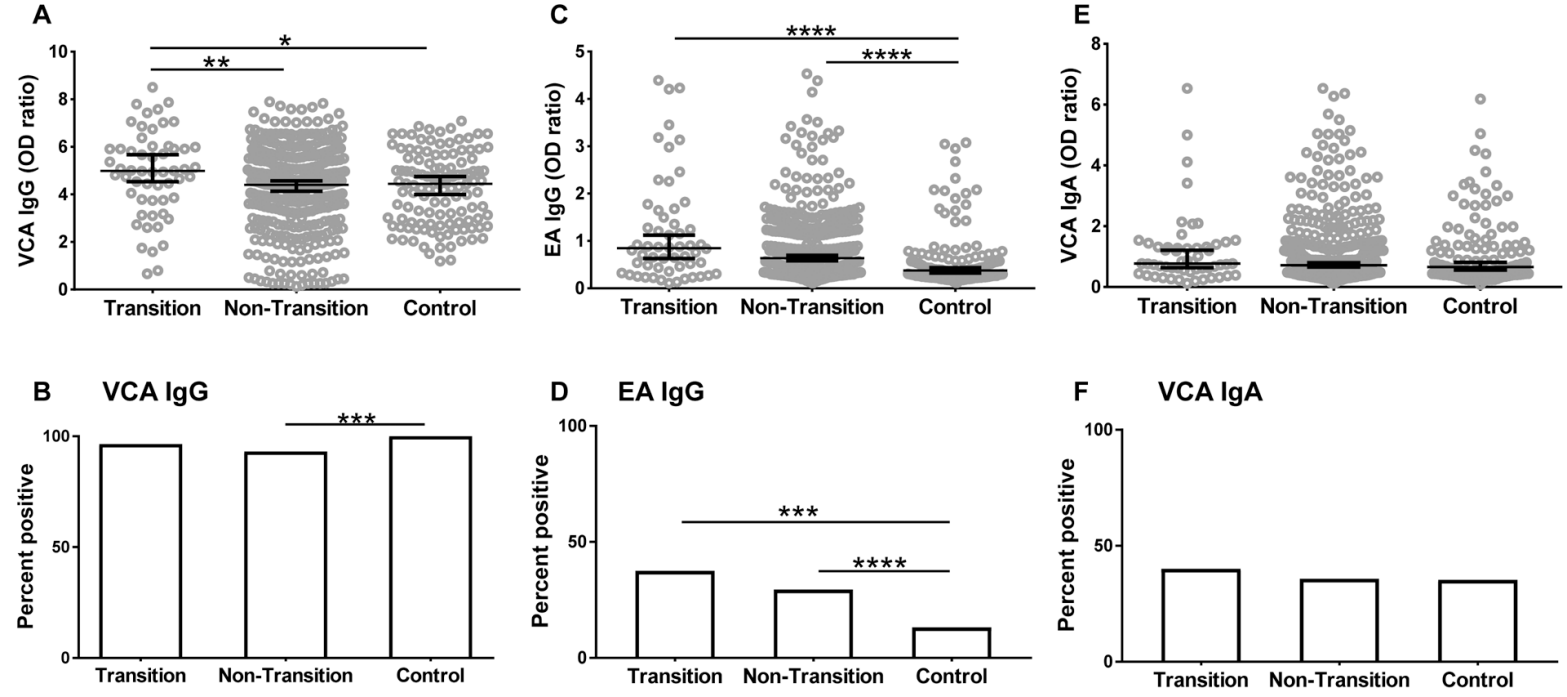
Relatives who subsequently transitioned to SLE had higher baseline levels of anti EBV IgG compared to healthy controls. IgG antibody levels against EBV VCA (A), EBV EA (C) and IgA antibodies against EBV VCA (E) were measured by ELISA at the baseline visit in relatives who transitioned to SLE (Transition), relatives who did not transition to SLE (Non-Transition), and unaffected unrelated controls (Control). Data are represented as median ± 95% CI. *p<0.05, **p<0.01, ****p<0.0001 by Mann Whitney. Seropositivity for VCA IgG (B), EA IgG (D), and VCA IgA (F) was determined as described in Methods. ***p<0.001, ****p<0.0001 by Fisher’s exact Test.

To understand the contribution of EBV reactivation to SLE classification, we compared the anti-EBV antibody levels at baseline visit in transitioned and non-transitioned relatives. Transitioned relatives had higher baseline VCA IgG levels compared to non-transitioned relatives (4.88±1.8 *vs* 4.18±1.81, *p*=0.0086, Figure 1A) and controls (4.88±1.8 *vs* 4.25±1.5, *p*=0.02). A higher percentage of controls were positive for VCA IgG compared to non-transitioned relatives (Figure 1B). However, VCA IgG seropositivity at baseline was not different between transitioned and non-transitioned relatives (Figure 1B), suggesting that both groups of SLE relatives had similar prior exposure to EBV. The total IgG concentrations were not different between transitioned and non-transitioned relatives, suggesting that the increase in VCA IgG levels is not due to hyperglobulinemia in the transitioned group (Supplementary figure S1).

At the baseline visit, the transitioned relatives had higher EA IgG levels compared to controls (1.19±1.11 *vs* 0.62±0.64, *p*<0.0001) (Figure 1C). Baseline EA IgG levels were not different between transitioned and non-transitioned relatives. A higher percentage of transitioned relatives were positive for EA IgG compared to controls (37.5% *vs* 13.11%, *p*<0.0005) but not compared to non-transitioned relatives (Figure 1D). IgA responses against VCA were not different between the groups (Figure 1E,F).

### Relatives who transitioned had higher levels of VCA IgG and EA IgG compared to ANA negative non-transitioned relatives

**Figure 2.**
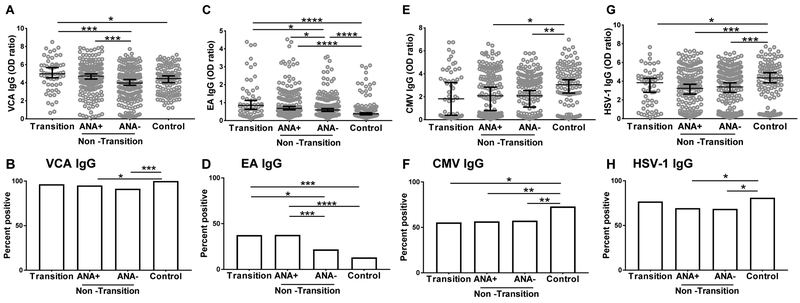
Relatives who transitioned to classified SLE had higher levels of VCA IgG and EA IgG compared to autoantibody negative non-transitioned relatives at baseline. Relatives who did not transition (Non-Transition) were divided based on their ANA positivity status (as described in Methods). Antibody levels (A,C,E,G) and seropositivity (B,D,F,H) for VCA IgG (A,B), EA IgG (C,D), CMV IgG (E,F), and HSV-1 (G, H) at the baseline visit were determined by ELISA. Data represented as median ± 95% CI. *p<0.05, **p<0.01, ***p<0.001, ****p<0.0001 by Mann Whitney (A,C,E,G) or Fisher’s exact Test (B,D,F,H).

A higher number of transitioned relatives were ANA positive at baseline compared to non-transitioned relatives and controls (Table 1). Both VCA IgG (r=0.15, *p*=0.002) and EA IgG levels (r=0.22, *p*<0.0001) correlated with increasing numbers of autoantibody specificities (Supplementary Table 1). To determine whether EBV serological reactivation contributes to ANA positivity, we divided the non-transitioned relatives into ANA positive (ANA+) and ANA negative (ANA-) groups at baseline. Transitioned relatives had significantly higher levels of VCA IgG compared to ANA- non-transitioned relatives (4.879±1.797 *vs* 3.866±1.795, *p*=0.0003 Figure 2A). Lower percentages of non-transitioned relatives were positive for VCA IgG at baseline compared to controls, irrespective of autoantibody positivity (Figure 2B).

Transitioned relatives had higher EA IgG at baseline compared to ANA- non-transitioned relatives (1.192±1.113 *vs* 0.7774±0.8484, *p*=0.0236, Figure 2C). Higher percentages of transitioned relatives were positive for EA IgG compared to ANA-, but not ANA+ non-transitioned relatives (Figure 2D). VCA IgG and EA IgG levels were higher in the ANA+ non-transitioned compared to ANA- non-transitioned relatives (Figure 2A,C); however, the levels of total IgG between these two groups were similar (Supplementary figure S1). These data suggest that increased EBV reactivation, as measured by EA IgG positivity and increased VCA IgG and EA IgG, may contribute to generation of autoantibodies, eventually contributing to autoimmunity.

Controls in this study had higher seropositivity and antibody levels against CMV and HSV-1 compared to transitioned and non-transitioned relatives (Figure 2E–H). However, the levels of CMV IgG and HSV-1 IgG were not different between transitioned and non-transitioned relatives, irrespective of ANA positivity. No differences in varicella virus (VZV) seropositivity or levels of VZV IgG were found between transitioned and non-transitioned relatives, nor with healthy controls (Supplemental Figure S2). These data show that the differences with VCA IgG and EA IgG seen between the transitioned and non-transitioned relatives are specific for humoral responses to EBV and not a generalized dysregulation of immune response to herpes viruses in SLE relatives.

### Increased levels of VCA IgG and EA IgG associate with transitioning to SLE

Our data show increased levels of VCA IgG and EA IgG at baseline prior to transitioning to classified disease in relatives of SLE patients. To determine whether these increased levels associate with transitioning to SLE after adjusting for age, sex, and race, we used GEE, accounting for correlation among families. Increasing levels of VCA IgG were associated with transitioning to SLE (Table 2).

Increased levels of VCA IgG remained significantly associated with transitioning to SLE after additionally adjusting the models for ANA positivity (OR 1.22 95%CI 1.01–1.47, *p*=0.04). EA IgG levels were also associated with transitioning to SLE (Table 2), when compared to non-transitioned relatives. No significant interactions were found between the anti-EBV antibody levels and age, sex, or race.

Increasing levels of VCA IgG and EA IgG were associated with transitioning to SLE when compared to controls (Table 2), and the association remained after additional adjustment for ANA positivity (OR 1.46, 95%CI 1.09–1.97, *p*=0.01 and OR 2.00 95%CI 1.21–3.31, *p*=0.007, respectively). Furthermore, EA IgG positivity was associated with transitioning to SLE (OR 4.35 95%CI 2.03–9.31, *p*=0.0002) when compared to controls. Levels of CMV IgG and HSV-1 IgG were not associated with transitioning to SLE (Table 2).

### Variants in IL10 and CD40 interact with anti-VCA antibodies in transitioning to SLE

Recent studies show a close association between environmental and host genetic factors in the development or exacerbation of the autoimmune response.[28 ,29] To test whether there is an association between immune response to EBV and variants in host genes implicated in EBV infection, we determined the interactions between variants in *IL10, CD40*, and *CR2* with anti-EBV responses in transitioning to SLE. We also tested interactions between anti-EBV responses and variants in *TNFAIP3*, a gene associated with SLE, but not implicated in EBV infection.

*IL10* variants were associated with transitioning to SLE (Table 3), however, no association was observed with the *CD40* variants or *CR2* variant in this small cohort.

**Figure 3.**
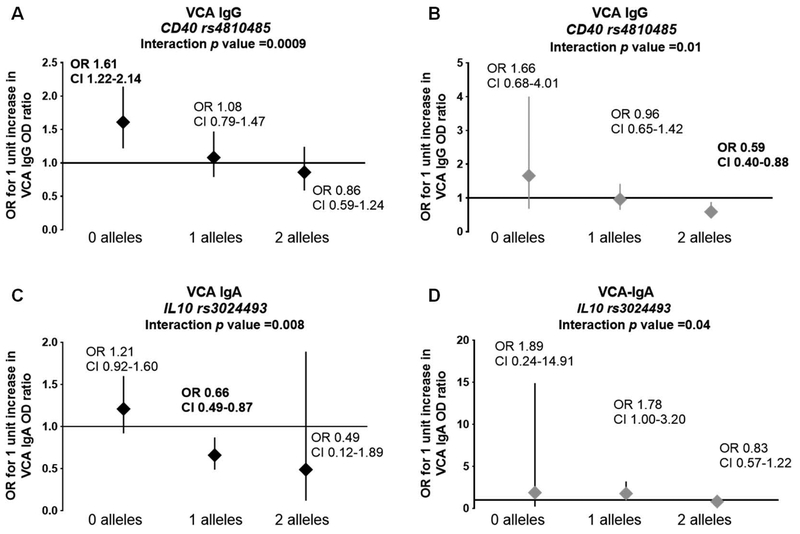
Significant interactions were observed between *CD40* variants and VCA IgG and between *IL10* variants and VCA IgA. (A, B) VCA IgG levels show significant interactions with *CD40* variant rs4810485 in the risk of transitioning to SLE. *CD40* rs4810485: Transition *n*=51, relatives who did not transition *n*= 355, unaffected unrelated controls *n*=115. A, interaction *p* value: *p*=0.0009 when compared to relatives who did not transition. B, interaction *p* value *p*=0.01, when compared to controls. (C,D) Significant interactions were observed between VCA IgA and *IL10* variant rs3024493 in the risk of transitioning to SLE. *IL10* rs3024493: Transition *n*=52, relatives who did not transition *n*=354, unrelated unaffected controls *n*=116. C, interaction *p* value = 0.008, when compared to those who did not transition, D, interaction *p* value *p*=0.04, when compared to controls.

The association between VCA IgG level and transitioning to SLE was modified by *CD40* rs4810485 (interaction *p=*0.0009) (Figure 3A). Increased VCA IgG level was associated with increased SLE risk in relatives with 0 minor alleles (OR=1.62, 95%CI 1.22–2.14), adjusting for age, sex, and race (Figure 3A). Similarly, the association between VCA IgA and transitioning to SLE was modified by *IL10* rs3024493 (interaction *p=*0.008) (Figure 3C). Although neither VCA IgA levels nor the SNP in *CR2* were associated with transitioning to SLE independently, we observed a modest but significant interaction between VCA IgA response and the *CR2* variant rs17615 in SLE transition risk (interaction *p=* 0.03) (Supplementary Table 2). Similar results were found when comparing transitioned relatives to controls (Figure 3B and 3D). We also tested association of SNPs in *TNFAIP3* as a gene associated with SLE, but not related to EBV. SNPs rs5029939 (in LD with rs5029937, rs2230926) and rs5029930 (in LD with rs3757173) were not associated with transitioning to SLE (Table 3). Furthermore, there were no significant interactions between TNFAIP3 SNPs and viral antibodies in transitioning to SLE.

## Discussion

In this study, we evaluated the contribution of EBV reactivation to transitioning to SLE in unaffected relatives of SLE patients. Our data show that SLE relatives had increased reactivation of EBV prior to transitioning to SLE, and increasing levels of EBV antibodies associated with SLE disease transitioning.

EBV infection has been linked to autoantibody production through molecular mimicry and epitope spreading. Aberrant antibody responses against Epstein-Barr Nuclear Antigen-1 (EBNA-1) can incite antibody responses against lupus autoantigens, Sm and Ro.[30 ,31] Anti-Ro responses were shown to be among the first autoantibodies detected preceding SLE classification in longitudinal samples.[32] In this study, we show that the transitioned relatives had increased VCA IgG and EA IgG responses compared to non-transitioned relatives who were ANA negative at baseline. There were significant differences between VCA IgG and EA IgG levels in the non-transitioned relatives based on their ANA positivity, and ANA+ relatives had significantly higher EBV antibody levels compared to ANA- relatives. In addition, differences in VCA IgA between transitioned and non-transitioned groups became significant when accounting for IL10 rs3024493. These data support that EBV reactivation may contribute to autoimmune responses through facilitating the loss of tolerance to lupus autoantigens. A prospective natural history study of high-risk individuals with serial evaluations and sample collection is needed to precisely define the timing of SLE transition relative to EBV reactivation and to perform a trajectory analysis over time.

We observed higher seroprevalence and higher levels of CMV IgG and HSV-1 IgG in unaffected controls compared to lupus relatives. CMV seroprevalance shows racial/ethnic differences[33] and is influenced by socioeconomic status.[34] The controls used in this study were recruited from community health centers where the exposure levels may be impacted by various factors including socioeconomic status. Levels of IgG directed against CMV and HSV-1 antigens did not show any significant differences between transitioned non-transitioned relatives. Furthermore, CMV IgG and HSV-1 IgG were not associated with transitioning to SLE. We also did not observe any differences in IgG responses towards varicella zoster (Supplementary Figure S2). The immune response to EBV is therefore unique in those who transitioned and is related to ANA positivity.

We observed increased levels of both VCA IgG and EA IgG in transitioned, as well as non-transitioned, SLE relatives when compared to controls. When transitioned relatives were compared to either non-transitioned relatives or controls, the association between VCA IgG levels and transitioning to SLE was modified by SLE-associated SNPs in *CD40* rs4810485 and VCA IgA levels were associated with increased risk of transitioning in individuals with 0 minor alleles at *IL10* rs3024493. These data suggest that the genetic predisposition to SLE, as is expected in relatives of SLE patients, influences the immune response to a latent EBV infection, and that the increased reactivation of EBV in the context of host genetic risk alleles may increase the risk of transitioning to SLE. However, the exact relationship between EBV antibodies and SLE risk alleles remains unclear. The presence of risk alleles may allow for a frequent reactivation of EBV through as yet unknown mechanisms.

Our data show that increased serological reactivation of EBV prior to SLE classification is associated with transitioning to SLE in genetically susceptible individuals. The negative predictive value for EA IgG positivity in SLE relatives was 89.33% and is possibly better than the currently available measures for assessing risk of transitioning in SLE relatives. We also show that there are significant interactions between SLE-associated variants in host genes implicated in viral-related pathways and measures of EBV serologic reactivation. To our knowledge, this is the first prospective study examining pre-clinical association between serologic measures of EBV reactivation and SLE disease transition.

## Supplementary Material

1S

2S

## Figures and Tables

**Table 1.** Study participant demographics^a^

Variable	Transitioned relatives n=56	Non transitioned relatives n=380	Unaffected Controls n=122	p-value
Age at baseline: mean (median) ± SD	47.4 (48.5) ± 12.1	47.2 (48.0) ± 15.8^b^	51.1 (51.0) ± 12.7	**0.01**
Time between re-contact (years): mean (median) ± SD	5.9 (5.1) ± 3.5	6.3 (5.4) ± 3.9^b^	NA^c^	0.50
Sex: Female (n,%)^d^	49 (87.5%)	316 (83.2%)	106 (86.9%)	0.49
Race (n,%)				**0.004**
European American	43 (76.8%)	279 (73.4%)	89 (73.0%)	
African American	9 (16.1%)	55 (14.5%)	15 (12.3%)	
Native American	4 (7.1%)	18 (4.7%)	9 (7.4%)	
Asian/Pacific Islander	0 (0.0%)	17 (4.5%)	5 (4.1%)	
Hispanic	0 (0.0%)	11 (2.9%)	4 (3.2%)	
ANA Positive Baseline (n,%)^e^	43 (76.8%)	183 (48.2%)	15 (12.3%)	**<0.0001**
Number of baseline ACR Criteria			ND^f^	**<0.0001**
0–1	7 (12.5%)	291 (76.5%)		
2	23 (41.1%)	74 (19.5%)		
3	26 (46.4%)	15 (4.0%)		

a p value for trend

b Not significant between transitioned and non-transitioned relatives by Mann-Whitney U test

c Not applicable

d Not significant between transitioned and non-transitioned relatives by Fisher’s exact test

e p<0.0001 between transitioned and non-transitioned relatives by Fisher’s exact test

f Not determined

**Table 2.** Increasing VCA IgG and EA IgG levels are associated with transitioning to SLE^a^

Serological measures	Transitioned (n=56) vs non transitioned (n=380) relatives OR (95% CI), p-value	Transitioned relatives (n=56) vs unrelated unaffected controls (n=122) OR (95% CI), p-value
VCA IgG (OD ratio)	**1.28 (1.07–1.53), 0.007**	**1.30 (1.02–1.65), 0.03**
VCA IgA (OD ratio)	0.99 (0.78–1.27), 0.94	1.01 (0.73–1.42), 0.91
EA IgG (OD ratio)	**1.43 (1.06–1.93), 0.02**	**2.11 (1.38–3.23), 0.0005**
CMV IgG (OD ratio)	1.03 (0.87–1.22), 0.72	0.89 (0.73–1.07), 0.21
HSV-1 IgG (OD ratio)	1.06 (0.92–1.22), 0.44	0.94 (0.82–1.09), 0.43

a Odds Ratio (OR) presented for 1 unit increase in OD ratio

**Table 3.** SNPs in *IL10* are associated with transitioning to SLE

Gene	SNP	Minor Allele	MAF	Transitioned to SLE (n=52) vs. Non Transition relatives (n=354) OR (95% CI) p-value	Transitioned (n=52) to SLE vs. Unaffected Controls (n=115) OR (95% CI) p-value
*IL10*	rs3024505	T	0.14	**2.15 (1.31–3.53) 0.002**	**1.87 (1.03–3.39) 0.04**
*IL10*	rs3024495	A	0.15	**2.13 (1.31–3.45) 0.002**	**2.00 (1.09–3.65) 0.02**
*IL10*	rs3024493	T	0.14	**2.06 (1.25–3.37) 0.004**	**1.91 (1.05–3.50) 0.03**
*IL10*	rs1800896	C	0.45	1.15 (0.77–1.72) 0.50	1.23 (0.74–2.06) 0.43
*IL10*	rs3122605	G	0.13	**1.86 (1.08–3.21) 0.02**	1.79 (0.98–3.27) 0.06
*CD40*	rs1569723	G	0.43	0.98 (0.72–1.33) 0.91	0.86 (0.59–1.23) 0.40
*CD40*	rs1883832	A	0.24	1.02 (0.62–1.68) 0.93	1.11 (0.63–1.94) 0.73
*CD40*	rs4810485	A	0.25	0.95 (0.57–1.59) 0.85	1.03 (0.59–1.80) 0.90
*CR2*	rs17615	T	0.31	0.97 (0.61–1.53) 0.88	0.72 (0.42–1.24) 0.24
*TNFAIP3*	rs5029930	C	0.14	1.41 (0.78–2.55) 0.25	1.49 (0.79–2.81) 0.22
*TNFAIP3*	rs5029939	G	0.05	1.19 (0.57–2.49) 0.65	1.96 (0.82–4.68) 0.13

## Key Messages

### What is already known about this subject?

- Significant associations between EBV serological markers and SLE have been shown
- SLE patients have higher frequencies of EBV infected cells, higher viral loads, and higher EA IgG levels

### What does this study add?

- This is a prospective study to understand the contribution of EBV reactivation prior to SLE disease onset in at risk individuals
- The study shows that previously unaffected relatives of SLE patients who subsequently develop classified SLE have higher serological reactivation of EBV compared to those who do not transition to SLE
- The study also shows that variants in genes that are associated with SLE and implicated in EBV infection, associate with transitioning to SLE

### How might this impact on clinical practice or future development?

- Serological measures of EBV reactivation may provide additional biomarkers to identify individuals at higher risk of transitioning to clinical SLE
