# Supplementary material for: Association of Epstein-Barr virus serological reactivation with transitioning to systemic lupus erythematosus in at risk individuals
Source: Ann Rheum Dis. Author manuscript; Available in PMC 2019 Sep 1. (PMC6692217; doi:10.1136/annrheumdis-2019-215361)
Supplement: 1S [file NIHMS1044057-supplement-1S.docx]

**SUPPLEMENTARY FIGURES**

**Association of Epstein-Barr virus serological reactivation with transitioning to systemic lupus erythematosus in at risk individuals**

Neelakshi R. Jog^1^, Kendra A. Young^2^, Melissa E. Munroe^1^, Michael T. Harmon^1^, Joel M. Guthridge^1^, Jennifer A. Kelly ^1^, Diane L. Kamen^3^, Gary S. Gilkeson^3^, Michael H. Weisman^4^, David R. Karp^5^, Patrick M. Gaffney ^1^, John B. Harley ^6, 7, 8^, Daniel J. Wallace^4^, Jill M. Norris^2^, Judith A. James^1, 9^

^1^Arthritis and Clinical Immunology Program, Oklahoma Medical Research Foundation, Oklahoma City, OK, USA

^2^Colorado School of Public Health, University of Colorado Anschutz Medical Campus, Aurora, CO, USA

^3^Department of Medicine, Medical University of South Carolina (MUSC), Charleston, SC, USA

^4^Division of Rheumatology, Cedar-Sinai Medical Center, Los Angeles, CA, USA

^5^Division of Rheumatic Diseases, University of Texas Southwestern Medical Center, Dallas, TX, USA

^6^Center of Autoimmune Genomics and Etiology, Cincinnati Children's Hospital Medical Center, Cincinnati, OH, USA

^7^Department of Pediatrics, University of Cincinnati College of Medicine, Cincinnati, OH, USA

^8^US Department of Veterans Affairs Medical Center, Cincinnati, OH, USA

^9^Departments of Medicine and Pathology, University of Oklahoma Health Science Center, Oklahoma City, OK, USA

**Correspondence:** Judith A. James, MD, PhD, Arthritis and Clinical Immunology, Oklahoma Medical Research Foundation, 825 NE 13^th^ Street, Oklahoma City, OK 73104, Phone: 405-271-4987, Fax: (405) 271-7063, Email: [judith-james@omrf.org](mailto:jamesj@omrf.org)

**Short title:** EBV sero-reactivation in lupus transition


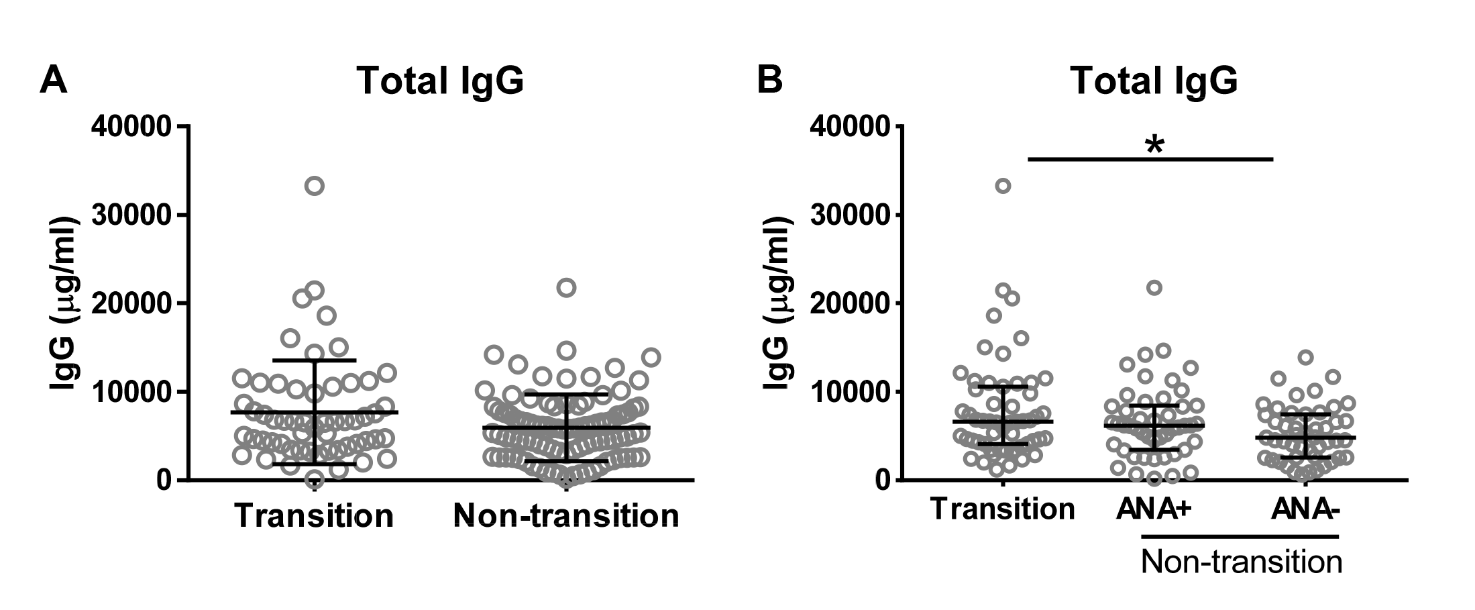


**Figure S1. Relatives who subsequently transitioned to SLE had similar levels of total IgG as baseline compared to those who did not transition.** Total IgG levels in serum were measured by ELISA at the baseline visit. A. Total IgG levels in relatives who transitioned to SLE (Transition, n=55) and matched relatives who did not transition to SLE (Non-Transition, n=94). B. Relatives who did not transition were divided based on ANA positivity status into ANA+ (n=47) and ANA- (n=47). Data are represented as median ± 95% CI. *p<0.05 by Mann Whitney.


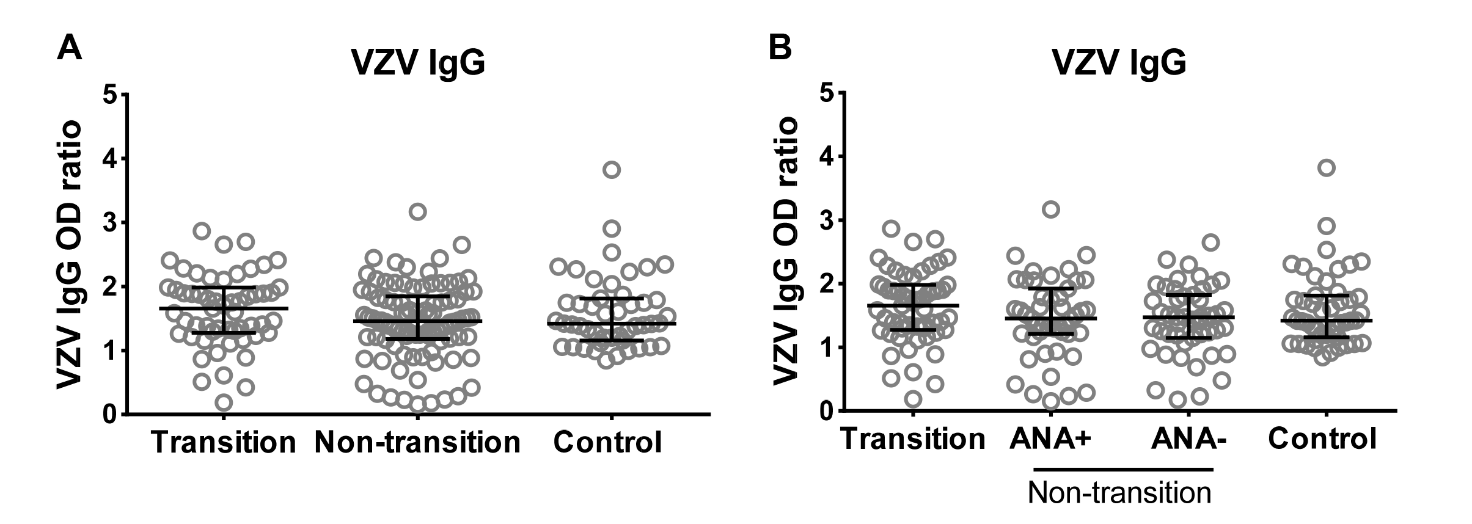


**Figure S2. Relatives who transitioned to classified SLE had similar levels of VZV IgG compared to relatives who did not transition and unrelated controls.** IgG responses towards Varicella zoster (VZV IgG) were measured by ELISA. A. VZV IgG levels at baseline in relatives who transitioned to SLE (Transition, n=55), matched relatives who did not transition to SLE (Non-Transition, n=94), and unaffected unrelated controls (Control n=47). B. into ANA+ (n=47) and ANA- (n=47). Data are represented as median ± 95% CI. p>0.05 by Mann-Whitney.
