# Supplementary material for: Association of Epstein-Barr virus serological reactivation with transitioning to systemic lupus erythematosus in at risk individuals
Source: Ann Rheum Dis. Author manuscript; Available in PMC 2019 Sep 1. (PMC6692217; doi:10.1136/annrheumdis-2019-215361)
Supplement: 2S [file NIHMS1044057-supplement-2S.docx]

**SUPPLEMENTARY TABLES**

**Association of Epstein-Barr virus serological reactivation with transitioning to systemic lupus erythematosus in at risk individuals**

Neelakshi R. Jog^1^, Kendra A. Young^2^, Melissa E. Munroe^1^, Michael T. Harmon^1^, Joel M. Guthridge^1^, Jennifer A. Kelly ^1^, Diane L. Kamen^3^, Gary S. Gilkeson^3^, Michael H. Weisman^4^, David R. Karp^5^, Patrick M. Gaffney ^1^, John B. Harley ^6, 7, 8^, Daniel J. Wallace^4^, Jill M. Norris^2^, Judith A. James^1, 9^

^1^Arthritis and Clinical Immunology Program, Oklahoma Medical Research Foundation, Oklahoma City, OK, USA

^2^Colorado School of Public Health, University of Colorado Anschutz Medical Campus, Aurora, CO, USA

^3^Department of Medicine, Medical University of South Carolina (MUSC), Charleston, SC, USA

^4^Division of Rheumatology, Cedar-Sinai Medical Center, Los Angeles, CA, USA

^5^Division of Rheumatic Diseases, University of Texas Southwestern Medical Center, Dallas, TX, USA

^6^Center of Autoimmune Genomics and Etiology, Cincinnati Children's Hospital Medical Center, Cincinnati, OH, USA

^7^Department of Pediatrics, University of Cincinnati College of Medicine, Cincinnati, OH, USA

^8^US Department of Veterans Affairs Medical Center, Cincinnati, OH, USA

^9^Departments of Medicine and Pathology, University of Oklahoma Health Science Center, Oklahoma City, OK, USA

**Correspondence:** Judith A. James, MD, PhD, Arthritis and Clinical Immunology, Oklahoma Medical Research Foundation, 825 NE 13^th^ Street, Oklahoma City, OK 73104, Phone: 405-271-4987, Fax: (405) 271-7063, Email: [judith-james@omrf.org](mailto:jamesj@omrf.org)

**Short title:** EBV sero-reactivation in lupus transition

**Supplementary Table 1. Increasing VCA IgG levels and EA IgG levels correlate with both increasing number of autoantibodies and ACR criteria**

|  | **Number of AutoAb**  **R (p value)** | **ACR score**  **R (p value)** |
| --- | --- | --- |
| VCA IgG OD ratio | **0.15 (0.002)** | **0.15 (0.002)** |
| VCA IgA OD ratio | 0.08 (0.08) | 0.008 (0.086) |
| EA IgG OD ratio | **0.22 (<0.0001)** | **0.15 (0.001)** |

**Supplementary Table 2. Significant interactions were found between variants in *IL10* and VCA IgA ISR and variants in *CD40* and VCA IgG ISR in the risk of transitioning to SLE. ^a^**

| **Gene** | **SNP** | **Antibody** | **Transitioned vs. Non Transition relatives**  **N=406**  **OR (95% CI)**  **p-value** | **Transitioned vs. Unaffected Controls**  **N=167**  **OR (95% CI)**  **p-value** | **Non Transition vs. Unaffected Controls**  **N=470**  **OR (95% CI)**  **p-value** |
| --- | --- | --- | --- | --- | --- |
| *IL10* | rs3024505 | VCA-IgG | 0.29 | 0.05 | 0.34 |
| *IL10* | rs3024505 | VCA-IgA | **0.01** | **0.047** | 0.82 |
| *IL10* | rs3024505 | EA | 0.35 | 0.60 | 0.82 |
| *IL10* | rs3024495 | VCA-IgG | 0.35 | 0.05 | 0.24 |
| *IL10* | rs3024495 | VCA-IgA | **0.03** | 0.11 | 0.77 |
| *IL10* | rs3024495 | EA | 0.22 | 0.59 | 0.86 |
| *IL10* | rs3024493 | VCA-IgG | 0.22 | **0.04** | 0.36 |
| *IL10* | rs3024493 | VCA-IgA | **0.008** | **0.04** | 0.81 |
| *IL10* | rs3024493 | EA | 0.18 | 0.40 | 0.66 |
| *IL10* | rs1800896 | VCA-IgG | 0.45 | 0.10 | 0.31 |
| *IL10* | rs1800896 | VCA-IgA | 0.55 | 0.92 | 0.65 |
| *IL10* | rs1800896 | EA | 0.30 | 0.24 | 0.14 |
| *IL10* | rs3122605 | VCA-IgG | 0.05 | 0.12 | 0.63 |
| *IL10* | rs3122605 | VCA-IgA | **0.02** | **0.005** | 0.41 |
| *IL10* | rs3122605 | EA | 0.70 | 0.97 | 0.81 |
| *CD40* | rs1569723 | VCA-IgG | **0.01** | **0.04** | 0.58 |
| *CD40* | rs1569723 | VCA-IgA | 0.16 | 0.69 | 0.40 |
| *CD40* | rs1569723 | EA | 0.10 | 0.21 | 0.12 |
| *CD40* | rs1883832 | VCA-IgG | **0.003** | **0.008** | 0.84 |
| *CD40* | rs1883832 | VCA-IgA | 0.46 | 0.79 | 0.71 |
| *CD40* | rs1883832 | EA | 0.09 | 0.40 | 0.22 |
| *CD40* | rs4810485 | VCA-IgG | **0.0009** | **0.01** | 0.48 |
| *CD40* | rs4810485 | VCA-IgA | 0.64 | 0.91 | 0.54 |
| *CD40* | rs4810485 | EA | 0.12 | 0.52 | 0.28 |
| *CR2* | rs17615 | VCA-IgG | 0.55 | 0.85 | 0.25 |
| *CR2* | rs17615 | VCA-IgA | **0.03** | 0.06 | 0.83 |
| *CR2* | rs17615 | EA | 0.99 | 0.25 | 0.67 |

^a^ Odds Ratio (OR) presented for 1 unit increase in OD ratio
